# Pelvic Organ Prolapse Is Associated with Osteoporosis in Korean Women: Analysis of the Health Insurance Review and Assessment Service National Patient Sample

**DOI:** 10.3390/jcm10163751

**Published:** 2021-08-23

**Authors:** Yoo-Ra Ko, Sa-Ra Lee, Sung-Hoon Kim, Hee-Dong Chae

**Affiliations:** Department of Obstetrics and Gynecology, Asan Medical Center, University of Ulsan College of Medicine, 88, Olympic-ro 43-gil, Songpa-gu, Seoul 05505, Korea; youzr90@gmail.com (Y.-R.K.); kimsung@amc.seoul.kr (S.-H.K.); hdchae@amc.seoul.kr (H.-D.C.)

**Keywords:** bone, connective tissue, menopause, osteoporosis, pelvic organ prolapse

## Abstract

Background and Objectives: Pelvic organ prolapse (POP) and osteoporosis are major disease entities in older women that have the same epidemiology and might also have the same molecular physiology. However, few data have been reported on the relationship between POP and osteoporosis. We designed this study to examine the association between POP and osteoporosis in Korean women. Materials and Methods: We used the Health Insurance Review and Assessment Service 2015 to 2017 National Patient Sample (HIRA-NPS). A total of 4,368,141 individuals were included in this study, and a total of 842,228 individuals aged 50 years and above were included in the final analysis. POP patients were defined by the Korean Informative Classification of Diseases (KOICD) codes (KCD-7, N81, or N99.3) and patients who underwent a pelvic reconstructive procedure. The osteoporosis patients were defined by KOICD (KCD-7, R4113, R3620, R0402,) who were prescribed osteoporosis medication. A 1:10 age-stratified matching and chi-squared test were used for statistical analysis, and *p* < 0.05 was considered as significant. Results: A total of 7359 women were included in this analysis. Advanced POP was correlated with osteoporosis in Korean women aged 50 years and above in 2015–2017 (*p* < 0.0001). After adjusting for age, advanced POP was correlated with osteoporosis in the 2015, 2016, and 2017 dataset (*p* = 0.013, 0.0009, 0.0119, respectively). Conclusions: Advanced POP is correlated with osteoporosis in Korean women aged 50 years and above. Evaluation for osteoporosis and education about bone health can be especially important, even in relatively young women, aged 50–59 years, and POP patients.

## 1. Introduction

Pelvic organ prolapse (POP) is defined as the abnormal descent or herniation of the pelvic organs (vagina, uterus, bladder, and/or rectum) from their normal position in the pelvis [1]. Osteoporosis is defined as a state of reduced bone density and quality that leads to an increased risk of fractures [2].

Both POP and osteoporosis have a high worldwide prevalence among aged women, occurring in 41% and 30% of women aged over 50 years, respectively [3,4]. In the Republic of Korea (ROK), the overall prevalence of POP is 31.7%, and patients aged over 50 years account for 57.5% of the cases [5]. Moreover, the prevalence of osteoporosis among women aged over 50 years in the ROK is reported to be 37.3% [6].

Previous studies have reported on the increasing trend of aging societies worldwide, and the ROK is one of the most rapidly aging countries. It has been predicted that the ROK will become a super-aged society in 2025, with the elderly population aged over 65 years accounting for 20.3% of the total population, which will increase to 43.9% by 2060 [7]. According to Statistics Korea, the proportion of people aged over 65 years increased from 13.1% of the Korean population in 2015 to 15.7% in 2020 [8]. In addition, the life expectancy of women is known to be longer than that of men [8]. Accordingly, the socioeconomic burden is especially higher in age-related diseases in women, including POP and osteoporosis. These two diseases can eventually lead to the deterioration in the quality of life of many women in the near future.

The known independent risk factors for POP include aging and parity, which can also be co-factors. However, the pathophysiology of POP is still unclear and is likely multifactorial [9]. POP is associated with the weakening of connective tissue and muscles in the pelvis and with neuromuscular dysfunction [10,11]. Insufficient collagen amount and changes in the extracellular matrix (ECM) of the pelvic floor are molecular changes that lead to the development of POP [12]. The co-occurrence of hypermobility of the joints, altered pulmonary compliance, and generalized connective tissue disorders include Marfan’s and Ehlers–Danlos syndromes, and POP supports this hypothesis [13,14].

Along with POP, bone strength, stability, and integrity are also affected by the quality of connective tissue [15]. An imbalance between bone resorption and formation leads to osteoporosis development [15]. The roles of the proteins and ECM that compose the bone matrix are also emerging as important elements [16]. The low estrogen level in aged women affects the mediators of the bone remodeling processes, which significantly increase bone resorption, thus accelerating bone loss [2].

POP and osteoporosis are major disease entities in older women who have the same epidemiology and might also have the same molecular physiology. Advanced age is a co-factor of both POP and osteoporosis, and menopause occurs with aging. Additionally, muscle weakness and poor quality of the ECM (especially collagen) worsen in a low estrogen environment. Therefore, considering the similarity in the pathophysiology of two diseases, we hypothesized that there is an association between menopause and an increased risk of POP and osteoporosis. Some studies have identified the relationship between POP and skeletal compromise in postmenopausal women [13,14,15,17,18,19,20]. However, to our knowledge, no large-scale studies have been conducted on the relationship between POP and osteoporosis. This study aimed to evaluate the relationship between advanced-stage POP and osteoporosis using recent data from a Korean nationally representative sample including over 840,000 Korean women.

## 2. Materials and Methods

### 2.1. Data Source

**Figure 1:**
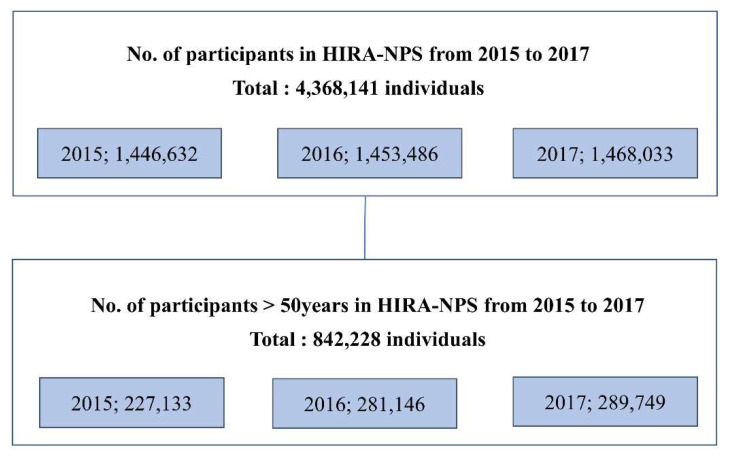
Study participants from the Health Insurance Review and Assessment Service National Patient Sample (HIRA-NPS) from 2015 to 2017.

We used the Health Insurance Review and Assessment Service National Patient Sample (HIRA-NPS), which includes approximately 4.3 million individuals, for evaluating the incidence of POP and osteoporosis from 2015 to 2017. The ROK has a universal health coverage system called the National Health Insurance Corporation. It covers approximately 98% of the total Korean population [21]. The HIRA evaluates the medical expenses charged by medical institutions to decide whether the costs are appropriate [22]. The HIRA has received claims made by all medical institutions in Korea since 2000, and the HIRA-NPS contains medical information such as age, sex, socioeconomic status, type of surgery, and prescription history [21]. As the data are newly extracted each year, individual patients’ data could not be continuously followed up. The diagnoses were coded per the International Classification of Diseases, 10th revision (ICD-10). The generic names of drugs were coded following the Korean national coding system. The HIRA-NPS, including approximately 1.4 million individuals, is a stratified random sample of 3% of the entire Korean population using 16 age groups and 2 sex groups. A total of 4,368,141 individuals were included in HIRA-NPS in 2015 (*n* = 1,446,632), 2016 (*n* = 1,453,486), and 2017 (*n* = 1,468,033). A total of 842,228 women (*n* = 271,333 in 2015, *n* = 281,146 in 2016, and *n* = 289,749 in 2017) aged over 50 years were included in the final analysis (Figure 1).

### 2.2. Study Design

The diagnosis was defined according to the Korean Standard Classification of Diseases, 7th edition (KCD-7), which was modified from the ICD-10. Patients with POP were defined using the Korean Informative Classification of Diseases (KOICD) codes (KCD-7: N81, female genital prolapse; N81.1, female cystocele; N81.2, incomplete uterovaginal prolapse; N81.3, complete uterovaginal prolapse; N81.4, uterovaginal pro-lapse, unspecified; N81.6, female rectocele; N81.8, other female genital prolapses; N81.9, female genital prolapse, unspecified; or N99.3, prolapse of the vaginal vault after a hysterectomy). Thereafter, we selected patients who underwent a pelvic reconstructive operation, as defined using the KOICD codes (KCD-7: R4113, insertion of a pessary; R3620, repair of cystocele; R0408, anterior colporrhaphy; R0410, posterior colporrhaphy; R0412, anterior and posterior colporrhaphy; R4202, vaginal hysterectomy; R4203, vaginal hysterectomy with anterior and posterior colporrhaphy; R04204, Manchester surgery; and Q3020, correction of rectocele) (Table 1).

Patients with osteoporosis were defined using the KOICD diagnostic codes (KCD-7: M80, osteoporosis with pathological fracture; M80.0, postmenopausal osteoporosis with pathological fracture; M80.1, post-oophorectomy osteoporosis with pathological fracture; M81.0, postmenopausal osteoporosis; and M82, osteoporosis in diseases classified elsewhere) based on dual-energy x-ray absorptiometry (DXA) examination (HC341–346) (Table 2). Among women who satisfied the above criteria, those who were taking antiresorptive agents including bisphosphonates, selective estrogen receptor modulators, denosumab, or anabolic agents including parathyroid hormone were included.

This study was performed using data from a deidentified secondary database, it was exempt from review by the institutional review board of Asan Medical Institute (exemption approval No. 2021-0755).

### 2.3. Statistical Analysis

All statistical analyses were conducted using SAS (version 9.4; SAS Institute Inc., Cary, NC, USA). To adjust for covariates, we used 1:10 age-stratified matching. The chi-square test was used for statistical analysis of the correlation between POP and osteoporosis. All statistical calculations were assumed to be statistically significant at *p* < 0.05.

## 3. Results

### Patients

**Figure 2:**
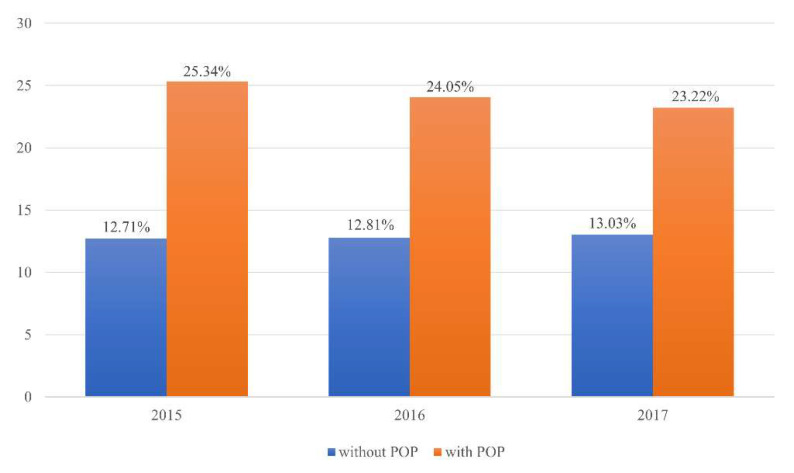
Prevalence of pelvic organ prolapse (POP) among women with osteoporosis, aged over 50 years (*p* < 0.0001).

In 2015, 2016, and 2017, the prevalence of osteoporosis among women with POP was 25.34%, 24.05%, and 23.22%, respectively, and these rates were statistically significantly higher than those in women without POP (*p* < 0.0001) (Figure 2) (Table 3).

**Figure 3:**
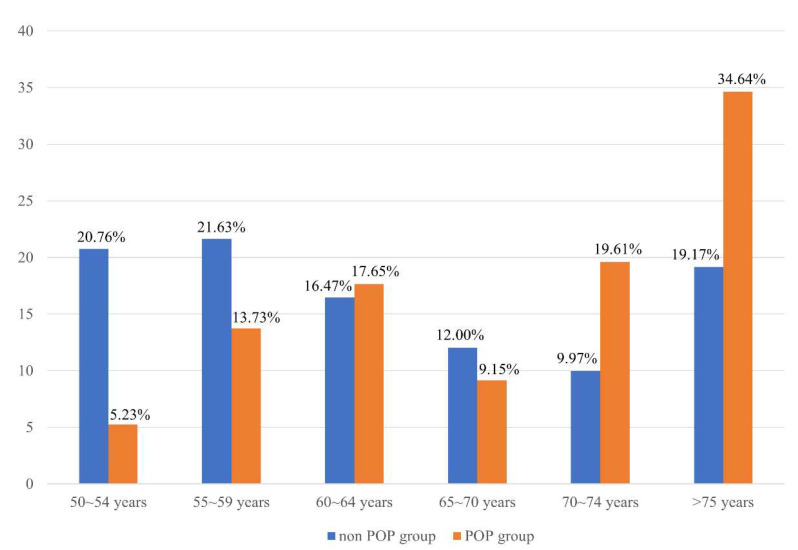
Increased prevalence of pelvic organ prolapse (POP) among women with osteoporosis (*p* < 0.0001).

As the age increased, the prevalence of POP increased among patients with osteoporosis. The prevalence of POP was 19.61% in the 70–74 years age group and 34.64% in the over 75 years age group. In contrast, younger patients (age 50–54 years) showed a lower prevalence of 5.23% (*p* < 0.0001) (Figure 3).

**Figure 4:**
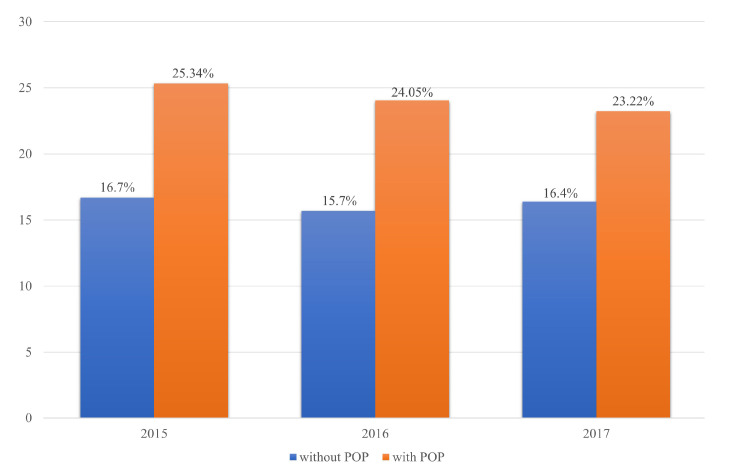
Prevalence of pelvic organ prolapse (POP) among women with osteoporosis aged over 50 years (1:10 age-stratified matching model; *p* = 0.013, 0.0009, and 0.0119 in 2015, 2016, and 2017, respectively).

After adjusting for age, the 1:10 age-stratified matching model showed a correlation between POP and osteoporosis in the 2015, 2016, and 2017 datasets (*p* = 0.013, 0.0009, and 0.0119, respectively) (Figure 4) (Table 4).

In the overall study period (2015–2017), the proportion of patients with POP and osteoporosis was 24.2%, and that of patients with POP but without osteoporosis was 24.02% (*p* = 0.0119) (Table 5).

## 4. Discussion

In this study, the prevalence of osteoporosis was significantly higher in the POP group than in the non-POP group of Korean women aged over 50 years.

The mechanisms underlying the association between POP and decreased bone mineral density (BMD) remain unclear. The high prevalence of POP and osteoporosis in postmenopausal women raises the possibility that the association is related to low estrogen levels and changes in the amount and quality of collagen and connective tissue [12,16]. Pelvic floor collagen is mainly composed of type I and III collagen, and type I collagen is also the principal component of the organic matrix of the bone [13]. Estrogen acts on collagen, which increases the strength of muscle and connective tissue, and on biochemical molecules that prevent the decomposition of collagen and elastin [23]. The hypoestrogenic environment of postmenopausal women alters the composition, structure, and catabolism of pelvic floor collagen, which leads to the weakening of pelvic supportive tissue [14,24]. The amounts of minerals and ECM (mainly type I collagen) in the bone determine its mechanical strength [25]. Estrogen also reduces the apoptosis of osteoblasts, which increases bone remodeling and formation and reduces bone resorption of osteoclasts, which leads to micro-architectural abnormalities of the bone [26]. Thus, low estrogen levels can affect bone quality and quantity, resulting in osteoporosis.

Previous studies have reported on the relationship between POP and osteoporosis. Pal et al. reported that moderate-to-severe POP is an independent and negative predictor of whole-body BMD in patients aged over 60 years in the analysis of the Women’ s Health Initiative Estrogen Plus Progestin trial (patients aged over 60 years, *n* = 11,089; moderate-to-severe POP/BMD tested, *n* = 958/46; no or mild POP/BMD tested, *n* = 10,127/634) [18]. The authors also reported that moderate-to-severe POP (any type) in postmenopausal women can be a risk factor for hip fractures (total, *n* = 15,760; moderate-to-severe POP, *n* = 1192; all fractures, *n* = 2156; hip fractures, *n* = 205) [19]. Our study results were similar to these reports in that we observed that osteoporosis was significantly more common in Korean women with advanced-stage POP requiring POP surgery. A recent study reported an association between pelvic floor disorder symptoms and bone strength in postmenopausal women [27]. Women with low bone quality had increased odds of urinary incontinence (any, urgency, mixed), whereas none of the pelvic floor disorder symptoms were associated with low bone quantity. Postmenopausal women with osteopenia had an increased risk of incontinence of solid stools compared with women with normal BMD. The authors also showed an increasing trend of fecal incontinence in women with osteoporosis, though there was no dose-related association [27].

The existence of a racial difference in POP and osteoporosis is well known. Therefore, the relationship between POP and osteoporosis should be clarified among women of the same race. Among Korean women, Lee et al. reported that the BMDs of the lumbar spine and femoral neck were not significantly different between the moderate-to-severe POP group and the no-to-mild POP group among 554 postmenopausal women aged 50–79 years [13]. However, the authors reported that women with severe POP had an increased risk of osteoporotic fractures, suggesting that the severity of POP can affect the relationship between POP and osteoporosis. This is similar to the results of our study, including women with advanced-stage POP requiring POP surgery.

In contrast with previous reports among elderly women aged over 60 years, a study in early postmenopausal women aged 55–60 years (*n* = 87) reported that the presence of POP is not helpful in predicting osteoporosis [20]. Therefore, the authors concluded that BMD does not need to be assessed in these relatively young postmenopausal women. This seems logical because age is one of the most important and obvious risk factors for osteoporosis, and whether the presence of POP is a stronger risk factor than age needs to be clarified.

This study has several strengths. First, our study represents the entire Korean population because of the use of the HIRA-NPS from the HIRA database [21]. The HIRA database contains 98% of Korean health insurance claims data, and this can be important in studies on diseases with racial differences, such as POP and osteoporosis. Second, this study included the largest number of patients in this setting. As shown above, several studies have reported inconsistent results on the relationship between POP and osteoporosis. This could be because of the lack of investigations with large patient populations. Third, our study analyzed patients with advanced-stage POP requiring surgery and patients with osteoporosis requiring medications other than postmenopausal hormone therapy.

However, this study had several limitations. First, as the data were separately extracted every year, continuous follow-up of individual patients was not possible. Second, because of the use of claims data, the accurate severity of POP (i.e., POP-Q staging) was not available. Therefore, we could not conclude a relationship between POP severity and osteoporosis. We can assume that the patients in this study had POP-Q stage III or IV because symptomatic POP requiring surgery has been mostly reported to be POP-Q stage III–IV, although in some cases, stage II POP can be included in symptomatic POP requiring surgery [28]. Third, personal characteristics proven to be risk factors for POP or osteoporosis, such as body mass index, physical activity, and parity, were not available. Further studies in patients with POP are needed to verify the results of this study. Fourth, the exact pathophysiological mechanism of the correlation between POP and osteoporosis remains unclear. Finally, we did not evaluate the relationship between osteoporotic fractures, the prevention of which is the final clinical goal of BMD evaluation, and POP.

The results of this study have implications for clinical practice, as they indicate that clinicians should consider evaluating BMD in women with POP aged over 50 years, although the current guidelines recommend performing DXA for BMD evaluation in postmenopausal women aged over 65 years.

## 5. Conclusions

Advanced POP has a relevant association with osteoporosis in Korean women aged over 50 years. Screening for osteoporosis and providing patient education about bone health should be considered in patients with POP, even in relatively young women in their 50 s.

## Figures and Tables

**Table 1:** Diagnostic codes for patients with pelvic organ prolapse, as defined by the Korean Informative Classification of Diseases.

N81	Female genital prolapse
N81.1	Female cystocele
N81.2	Incomplete uterovaginal prolapse
N81.3	Complete uterovaginal prolapse
N81.4	Uterovaginal prolapse, unspecified
N81.6	Female rectocele
N81.8	Other female genital prolapse
N81.9	Female genital prolapse, unspecified
N99.3	Prolapse of the vaginal vault after a hysterectomy
R4113	Insertion of a pessary
R3620	Repair of cystocele
R0408	Anterior colporrhaphy
R0410	Posterior colporrhaphy
R0412	Anterior and posterior colporrhaphy
R04202	Vaginal hysterectomy
R04203	Vaginal hysterectomy with anterior and posterior colporrhaphy
R04204	Manchester surgery
Q3020	Correction of rectocele

**Table 2:** Diagnostic codes for patients with osteoporosis, as defined by the Korean Informative Classification of Diseases.

M80	Osteoporosis with pathological fracture
M80.0	Postmenopausal osteoporosis with pathological fracture
M80.00	Postmenopausal osteoporosis with pathological fracture, multiple sites
M80.01	Postmenopausal osteoporosis with pathological fracture, shoulder region
M80.02	Postmenopausal osteoporosis with pathological fracture, upper arm
M80.03	Postmenopausal osteoporosis with pathological fracture, forearm
M80.04	Postmenopausal osteoporosis with pathological fracture, hand
M80.05	Postmenopausal osteoporosis with pathological fracture, pelvic region and thigh
M80.06	Postmenopausal osteoporosis with pathological fracture, lower leg
M80.07	Postmenopausal osteoporosis with pathological fracture, ankle and foot
M80.08	Postmenopausal osteoporosis with pathological fracture, other
M80.09	Postmenopausal osteoporosis with pathological fracture, site unspecified
M80.1	Postoophorectomy osteoporosis with pathological fracture
M80.10	Postoophorectomy osteoporosis with pathological fracture, multiple sites
M80.11	Postoophorectomy osteoporosis with pathological fracture, shoulder region
M80.12	Postoophorectomy osteoporosis with pathological fracture, upper arm
M80.13	Postoophorectomy osteoporosis with pathological fracture, forearm
M80.14	Postoophorectomy osteoporosis with pathological fracture, hand
M80.15	Postoophorectomy osteoporosis with pathological fracture, pelvic region and thigh
M80.16	Postoophorectomy osteoporosis with pathological fracture, lower leg
M81.0	Postmenopausal osteoporosis
M82.0	Osteoporosis in diseases classified elsewhere

**Table 3:** Pelvic organ prolapse (POP) and osteoporosis prevalence in women aged over 50 years, as defined by the Korean Informative Classification of Diseases.

**Year, 2015**	**Without Osteoporosis**	**With Osteoporosis**	**Total**
Without POP, *n* (%)	236,665 (87.29%)	34,447 (12.71%)	271,112
With POP, *n* (%)	165 (74.66%)	56 (25.34%)	221
χ^2^ test < 0.0001	236,830	34,503	271,333
**Year, 2016**	**Without Osteoporosis**	**With Osteoporosis**	**Total**
Without POP, *n* (%)	244,919 (87.19%)	35,990 (12.81%)	271,112
With POP, *n* (%)	180 (75.95%)	57 (24.05%)	221
χ^2^ test < 0.0001	245,099	36,047	281,146
**Year, 2017**	**Without Osteoporosis**	**With Osteoporosis**	**Total**
Without POP, *n* (%)	251,797 (86.97%)	37,741 (13.03%)	289,538
With POP, *n* (%)	162 (76.78%)	49 (23.22%)	221
χ^2^ test < 0.0001	251,959	37,790	289,749

**Table 4:** Pelvic organ prolapse (POP) and osteoporosis prevalence in women aged over 50 years (1:10 age-stratified matching model).

**Year, 2015**	**Without Osteoporosis**	**With Osteoporosis**	**Total**
Without POP, *n* (%)	1841 (83.3%)	369 (16.7%)	2210
With POP, *n* (%)	165 (74.66%)	56 (25.34%)	221
χ^2^ test = 0.0013	2006	425	2431
**Year, 2016**	**Without Osteoporosis**	**With Osteoporosis**	**Total**
Without POP, *n* (%)	1998 (84.3%)	372 (15.7%)	2370
With POP, *n* (%)	180 (75.95%)	57 (24.05%)	237
χ^2^ test = 0.0009	2178	429	2607
**Year, 2017**	**Without Osteoporosis**	**With Osteoporosis**	**Total**
Without POP, *n* (%)	1764 (83.6%)	346 (16.4%)	2110
With POP, *n* (%)	162 (76.78%)	49 (23.22%)	221
χ^2^ test = 0.0119	1926	395	2321

**Table 5:** Pelvic organ prolapse (POP) and osteoporosis prevalence in women aged over 50 years in 2015–2017 (1:10 age-stratified matching model).

Year, 2015–2017	Without Osteoporosis	With Osteoporosis	Total
Without POP, *n* (%)	733,381 (87.1%)	108,178 (12.9%)	841,559
With POP, *n* (%)	507 (75.8%)	162 (24.2%)	669
χ^2^ test = 0.0119	733,888	108,340	842,228

## Data Availability

The Excel data used to support the findings of this study were supplied by Sa Ra Lee under license, and requests for access to these data should be made to S.-R.L. leesr@amc.seoul.kr.
